# Reducing Cabozantinib Toxicity in Renal Cell Carcinoma Treatment through Structural Modifications

**DOI:** 10.2174/0115734064374511250411104320

**Published:** 2025-04-18

**Authors:** Jiaxiang Guo, Xiaotao Yin, Yongliang Lu, Yu Yang

**Affiliations:** 1 Department of Urology, the Fourth Medical Center of PLA General Hospital, Beijing 100853, China

**Keywords:** SARs, Renal Cell Carcinoma (RCC), Tyrosine Kinase Inhibitors (TKIs), cabozantinib, target profiling, toxicity

## Abstract

**Background and Objectives:**

Cabozantinib, a Tyrosine Kinase Inhibitor (TKI), is widely used in Renal Cell Carcinoma (RCC) therapy but often causes serious side effects such as myelosuppression, immunosuppression, and angiopathy. This study aims to identify key protein targets responsible for the therapeutic efficacy and adverse reactions of cabozantinib and to explore structural modifications to reduce toxicity while preserving efficacy.

**Methods:**

A non-randomized computational approach was employed, screening 400 potential protein targets using SwissTargetPrediction and ChemBL databases. Molecular docking and Structure-Activity Relationship (SAR) analysis were performed to assess interactions between cabozantinib and identified targets, focusing on structural elements contributing to toxicity.

**Results:**

Three primary proteins were identified as responsible for the anti-tumor effects of cabozantinib, while three others were linked to its side effects. Docking analysis revealed that the methoxyphenyl group in cabozantinib formed undesirable hydrogen bonds with toxicity-related proteins. Modulating these off-target interactions by minimizing hydrogen bonding in this region could significantly reduce adverse effects.

**Conclusion:**

These findings provide structural insights into cabozantinib’s dual effects and suggest optimization strategies for TKI design, offering a pathway toward safer and more effective RCC treatments.

## 1 INTRODUCTION

Renal cancer remains a challenging disease to treat, with many patients enduring severe side effects from current therapeutic options [1-3]. Researchers have traditionally focused on optimizing drug-target binding efficacy to enhance treatment outcomes [4-6]. However, one key challenge often overlooked is the dynamic nature of blood flow, which facilitates rapid drug metabolism [7, 8]. This accelerated breakdown of therapeutic agents can lead to off-target effects and the accumulation of toxic metabolites, significantly contributing to the adverse side effects experienced by patients [9].

Renal Cell Carcinoma (RCC) has a variety of targeted therapies available, but as outlined in Fig. (**1**), four key drugs, including cabozantinib, have become the mainstay in current treatment strategies [10, 11]. These drugs primarily target critical receptors involved in angiogenesis, such as Vascular Endothelial Growth Factor Receptor (VEGFR) and Platelet-Derived Growth Factor Receptor (PDGFR), to inhibit tumor vascularization and growth [12]. However, despite their intended specificity, these drugs can also interact with other protein targets throughout the body [13-15]. This off-target binding contributes to a range of toxic side effects, as these unintended interactions disrupt normal cellular functions in non-cancerous tissues [16-18].

The use of multi-targeted drugs has also introduced a broader range of toxic side effects, including bone marrow suppression, immune suppression, hypertension, bleeding risks, fatigue, and gastrointestinal toxicity [14, 19-21]. These adverse effects arise from the interaction of the drugs with multiple biological targets beyond their primary intended pathways [19, 22]. Through reverse prediction from structure to target, we have identified several additional potential targets for these drugs [23-25]. The discovery of these new targets opens up opportunities for researchers to refine drug design strategies [26-30]. By considering these alternative targets during the development process, future therapies could be engineered to minimize off-target effects while maximizing therapeutic efficacy, leading to more precise and safer treatment options [31-34].

## 2 MATERIALS AND METHODS

### 2.1 Screening of Anti-renal Cancer Protein Targets of Four Compounds

In this study, we utilized the SWISS database to identify potential protein targets associated with Tyrosine Kinase Inhibitors (TKIs) [35-37]. A comprehensive screening process was conducted, resulting in the selection of target proteins relevant to these compounds. This selection aimed to capture a broad spectrum of potential interactions, providing a foundation for further analysis of the therapeutic and adverse effects linked to TKIs [38, 39]. The identified targets will facilitate a deeper understanding of the mechanisms through which these drugs exert their anti-tumor effects in the context of renal cancer treatment.

### 2.2 Intersection of Anti-renal Cancer Protein Targets of Four Compounds

In our study, we utilized the online tool available at “https://bioinfogp.cnb.csic.es/tools/venny/index.html” to identify and analyze potential protein targets associated with Tyrosine Kinase Inhibitors (TKIs) [40-42]. Initially, we screened a comprehensive database to generate a list of 400 potential target proteins relevant to these compounds. This screening process involved a systematic evaluation of the top predicted target proteins for the selected TKIs, allowing us to intersect and compare the target profiles [43-45]. The resulting intersection highlighted a set of common target proteins, which are crucial for further exploration of their roles in mediating the therapeutic effects and potential side effects associated with these inhibitors. This methodological approach facilitates a deeper understanding of the multi-target interactions that characterize TKIs in the context of renal cancer treatment.

### 2.3 Toxicity and Corresponding Protein Targets

Using the ChemBL database, we further categorized the identified targets from the SWISS database [38, 41, 46-49]. This analysis led to the identification of three primary proteins through which Tyrosine Kinase Inhibitors (TKIs) exert their anti-renal cancer effects [50, 51]. Additionally, we discovered three key proteins associated with the adverse side effects of these drugs. This classification enhances our understanding of the therapeutic mechanisms and potential toxicities related to TKIs in renal cancer treatment, providing a valuable framework for future drug development and safety assessments.

### 2.4 Molecular Docking of Cabozantinib with Primary Renal Cancer Targets to Reduce Toxicity

To investigate the targets associated with the side effects of Tyrosine Kinase Inhibitors (TKIs), we utilized the PDB protein database for target screening [50-58]. Subsequently, we employed Discovery Studio to analyze the Structure-Activity Relationship (SAR) of these targets in conjunction with cabozantinib. This analysis included a comparative assessment with the SAR of the identified anti-renal cancer targets, allowing us to pinpoint critical structural optimization sites within cabozantinib. This approach aims to enhance the therapeutic efficacy of cabozantinib while mitigating its adverse effects.

## 3 RESULTS AND DISCUSSION

### 3.1 Screening of Anti-renal Cancer Protein Targets of Four Compounds

**Fig. (2):**
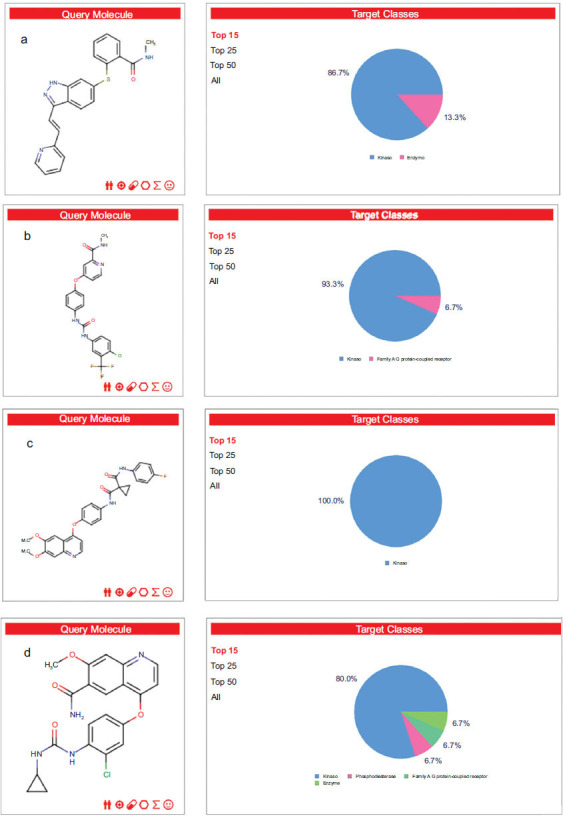
Target classes of four compounds: **a**) Sorafenib, **b**) Axitinib, **c**) Cabozantinib, and **d**) Lenvatinib.

Using the SwissTargetPrediction database [59-62], we screened 400 potential protein targets for the four compounds under investigation (Fig. **2**). Among the identified targets, kinase proteins were the predominant category. Kinases are critical regulators of various cellular signaling pathways, including those responsible for tumor growth, angiogenesis, and metastasis [63-68]. Given their central role in renal cancer progression, targeting these kinases provides a strategic approach for therapeutic intervention, highlighting their importance in the design and development of anti-cancer drugs for renal cell carcinoma.

### 3.2 Intersection of Anti-renal Cancer Protein Targets of Four Compounds

We utilized Venny 2.1.0 (csic.es) to identify the common targets among the screened proteins in Fig. (**3**), resulting in a total of seven proteins, including VEGFR. Notably, in Table **1**, three of these proteins: Serine/threonine-protein kinase Aurora-B, LCK (Tyrosine-protein kinase LCK), and TEK (Tyrosine-protein kinase TIE-2) are likely to be the primary targets associated with the observed toxic side effects. These proteins are integral to various cellular processes, and their interaction with the compounds may contribute significantly to the adverse effects experienced by patients undergoing treatment for renal cell carcinoma.

### 3.3 Toxicity and Corresponding Protein Targets

The four drugs in question are multi-target Tyrosine Kinase Inhibitors (TKIs) primarily used for treating Renal Cell Carcinoma (RCC) by inhibiting various signaling pathways related to angiogenesis and tumor growth. However, they may also indirectly affect these proteins through other mechanisms or cross-target interactions, leading to shared toxic side effects associated with these targets. As shown in Table **2**, AURKB is involved in regulating cell division and chromosome distribution [69-71]. Its inhibition can adversely affect rapidly dividing bone marrow cells, resulting in leukopenia (increased infection risk), anemia (reduced red blood cells), and thrombocytopenia (increased bleeding risk) [72, 73]. LCK is a crucial signaling molecule for T-cell activation. Its inhibition can weaken T-cell activity, impair immune response, and raise the risk of infections, particularly viral, bacterial, and fungal infections [74]. TEK primarily regulates angiogenesis and vascular function in endothelial cells [36]. Inhibiting angiogenic signaling pathways may lead to abnormal vascular regulation, often resulting in hypertension, a common issue among patients treated with TKIs. Therefore, we aim to investigate the interactions of these three proteins and explore the structure-activity relationship of cabozantinib to find a balance that maintains molecular activity while reducing side effects.

### 3.4 Molecular Docking of Cabozantinib with Primary Renal Cancer Targets to Reduce Toxicity

To elucidate the structural causes behind the toxic side effects presented by these four drugs, we conducted a thorough target screening. Key binding sites, AURKB (PDB ID: 4AF3), LCK (PDB ID: 3LCK), and TEK(PDB ID: 2GY5) were identified. These sites have been recognized as critical in mediating the toxicities commonly seen with targeted anti-cancer therapies. Subsequently, we performed molecular docking for each drug against these targets, aiming to uncover the structural basis for adverse effects. The results demonstrated libdock scores above 85 for all four compounds, as shown in Table **3**, indicating strong binding affinity with these proteins. This suggests that interactions at these sites are likely contributors to the myelosuppression, immunosuppression, and angiopathy frequently observed with cabozantinib and related Tyrosine Kinase Inhibitors (TKIs).

To retain the therapeutic efficacy of cabozantinib while minimizing its toxic side effects, we conducted molecular docking studies focused on cabozantinib’s primary renal cancer targets: VEGFR2 (PDB ID: 3VO3), MET (PDB ID: 3DKC), RET (PDB ID: 2IVU), and Trk-A (PDB ID: 6PL3). These docking experiments were aimed at elucidating the interactions between cabozantinib and these key proteins. As shown in Table **4**, the docking scores for each target exceeded 100, indicating robust binding affinity. These findings provide insights into cabozantinib’s mechanism of action at its main therapeutic targets, which may inform strategies to optimize its efficacy while reducing off-target toxicities.

### 3.5 Structure-Activity Relationship Analysis of Cabozantinib with Renal Cancer Treatment and Toxicity-Associated Targets

To further understand the balance between therapeutic efficacy and potential toxicity, we analyzed the Structure-Activity Relationships (SAR) of cabozantinib with six key protein targets: three associated with toxic side effects (4AF3, 3LCK, 2GY5) and three primary anti-tumor targets (AURKB, LCK, TEK, and Trk-A).

As shown in Figs. (**4** and **5**), cabozantinib’s interaction with 4AF3 reveals notable hydrogen bonding in the 2D interaction diagram between two phenoxy groups of cabozantinib and residues LYS168 and GLU161. In the 3D diagram, these phenoxy groups are embedded within the protein, suggesting their critical role in stabilizing the interaction with 4AF3. In the interaction with 3LCK, one phenoxy group of cabozantinib forms strong hydrogen bonds with residues LYS379, GLU317, LEU300, and HIS297, while the benzene ring of the phenoxy moiety exhibits a Pi-Sigma interaction with ARG302, highlighting this structure's importance in binding affinity. Similarly, cabozantinib’s interaction with 2GY5 shows hydrogen bonding between one phenoxy group and residues ARG50, TRP49, PRO388, and VAL421, with a notable Pi-Pi T-shaped interaction between the benzene ring and TRP82. This structural embedding, especially in the 3D interaction views, reinforces the pivotal role of these moieties in binding to toxicity-related targets.

We further explored the stability ofthe binding interactions of cabozantinib with the three toxicity-related protein targets (4AF3, 3LCK, and 2GY5) through molecular dynamics simulations. These simulations revealed that cabozantinib maintains stable interactions with each of these proteins within a 10-picometer range. This highlights hydrogen bonding as the primary binding interaction for cabozantinib with these proteins, supporting the critical role of these bonds in anchoring cabozantinib to toxicity-associated sites.

In our investigation of cabozantinib’s interactions with critical renal cancer targets, namely VEGFR2 (PDB ID: 3VO3), MET (PDB ID: 3DKC), RET (PDB ID: 2IVU), and Trk-A (PDB ID: 6PL3), we observed several key findings. As illustrated in Fig. (**6**), the 2D interaction diagram reveals that the phenoxy structure of cabozantinib exhibits minimal interaction with the RET target (2IVU), while the corresponding 3D representation shows the phenoxy moiety positioned outside the binding site, indicating a lack of interaction. For the MET target (3DKC), the 2D diagram shows a weak Pi-Anion interaction between the phenoxy structure and residues ASP1222 and Mg^2+^. However, the 3D visualization further confirms that the phenoxy structure remains outside the binding site, signifying no significant interaction. Similarly, in the case of VEGFR2 (3VO3), the 2D interaction only indicates a weak Pi-Alkyl interaction with residue LEU1035, and the 3D diagram reaffirms that the phenoxy moiety is located outside the binding pocket. The 2D interaction analysis indicates that Trk-A (PDB ID: 6PL3) has a weak Pi-Cation interaction with residue GIY 670. The 3D visualization further confirms that the phenoxy structure does not occupy the key binding site of this target. Based on these observations, we conclude that optimizing the structure of cabozantinib’s phenoxy group to reduce its electronegativity may mitigate the associated toxicity while retaining its therapeutic efficacy. This structural refinement could enhance the drug's safety profile, making it a valuable approach in future drug development.

## CONCLUSION

This study underscores the intricate balance between the therapeutic efficacy and potential side effects of Tyrosine Kinase Inhibitors (TKIs), with a particular focus on cabozantinib in the treatment of Renal Cell Carcinoma (RCC). By leveraging computational modeling, target screening, and docking analyses, we identified key protein targets that influence both the anti-tumor activity and toxicity profile of cabozantinib. Through molecular docking and molecular dynamics simulations, we demonstrated that the methoxyphenyl (anisole) group in cabozantinib plays a critical role in its off-target interactions. Specifically, strong hydrogen bonding interactions were observed between this group and three key toxicity-related proteins: AURKB (PDB ID: 4AF3), LCK (PDB ID: 3LCK), and TEK (PDB ID: 2GY5), which are implicated in adverse effects such as myelosuppression, immunosuppression, and angiopathy. These findings suggest that this structural feature may significantly contribute to cabozantinib’s toxic side effects. In contrast, docking analyses with four key anti-tumor targets: VEGFR2 (PDB ID: 3VO3), MET (PDB ID: 3DKC), RET (PDB ID: 2IVU), and Trk-A (PDB ID: 6PL3) revealed only weak interactions, including Pi-Anion, Pi-Alkyl, and Pi-Cation interactions, rather than strong hydrogen bonding. This suggests that the methoxyphenyl group is not a critical determinant of cabozantinib’s anti-tumor activity. Consequently, structural modification of this region, such as replacing or removing the methoxy substituent is unlikely to compromise the drug’s therapeutic effects but could substantially reduce its toxicity by minimizing off-target interactions.

Our findings highlight the potential for structure-based drug optimization to enhance the safety profile of cabozantinib. Rational drug design approaches, such as eliminating or modifying the methoxyphenyl moiety, could lead to the development of next-generation TKIs with a higher therapeutic index. This is particularly crucial for RCC patients undergoing long-term chemotherapy, as reducing drug-induced adverse effects could improve patient adherence, quality of life, and overall clinical outcomes. Despite these promising insights, several limitations must be acknowledged. While our computational approach provides valuable structural and interaction-based predictions, experimental validation is required to confirm the impact of methoxyphenyl modifications on both efficacy and toxicity. Future studies should include the synthesis and bioassay evaluation of cabozantinib analogs, assessing their biological activity against RCC cells, and evaluating their toxicity profiles. Additionally, advanced molecular simulations, such as Free Energy Perturbation (FEP) or Quantum Mechanics/Molecular Mechanics (QM/MM) simulations, could provide deeper insights into the energetic contribution of the methoxyphenyl group to binding affinity.

Further research should also explore the pharmacokinetic and toxicological impact of structural modifications, particularly their influence on drug metabolism, bioavailability, and systemic toxicity. Evaluating potential off-target effects in different organ systems, such as the hematopoietic and immune systems, will be crucial for translating these findings into clinical applications. Moreover, applying similar structural optimization strategies to other multi-target TKIs may provide a broader framework for enhancing drug selectivity and minimizing adverse effects. This study underscores the importance of comprehensive target profiling and rational drug design in improving cancer therapies. By carefully balancing efficacy and safety, our findings pave the way for more precise, patient-friendly treatments for RCC and other malignancies. Future research integrating experimental validation and pharmacological optimization will be essential for translating these insights into tangible clinical benefits.

## Figures and Tables

**Fig. (1):**
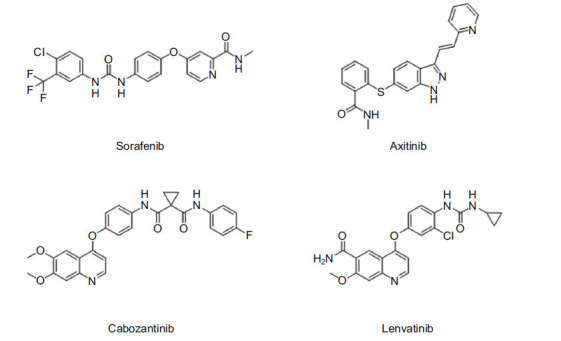
Targeted drugs against kidney cancer.

**Fig. (3):**
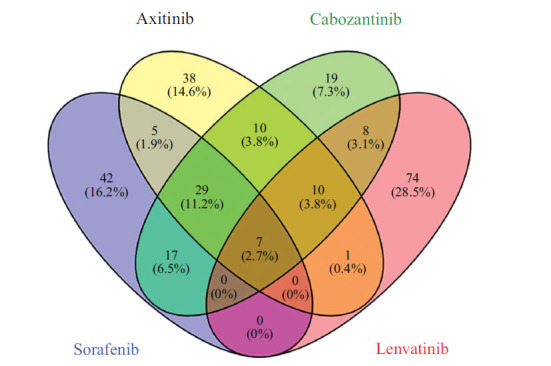
Venn diagram showing the targets of the four compounds.

**Fig. (4):**
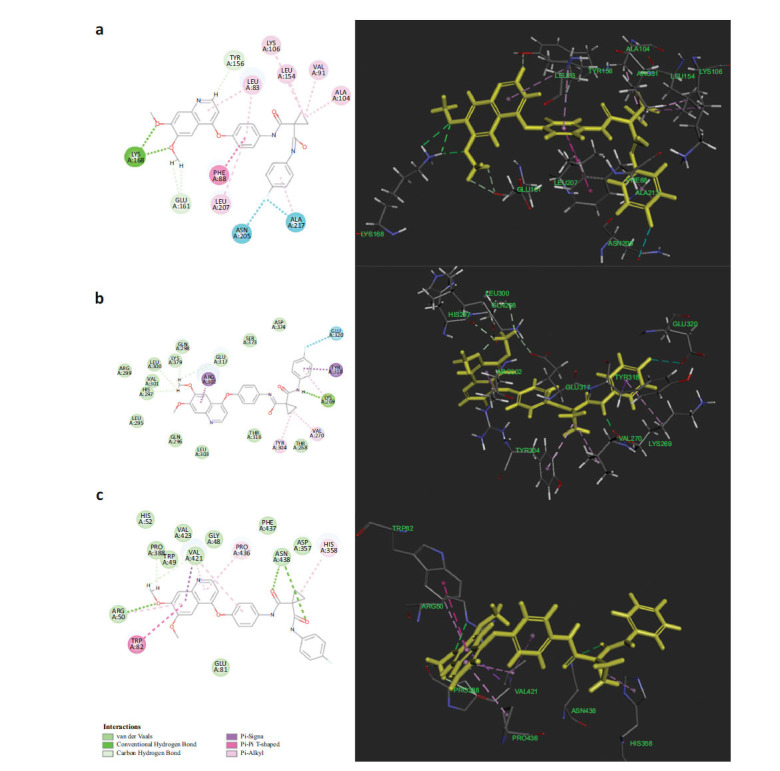
2D and 3D interaction diagrams of cabozantinib with three toxicity-associated targets. Left: 2D interaction maps; Right: 3D interaction structures. (**a**) AURKB (PDB ID: 4AF3), (**b**) LCK (PDB ID: 3LCK), and (**c**) TEK(PDB ID: 2GY5).

**Fig. (5):**
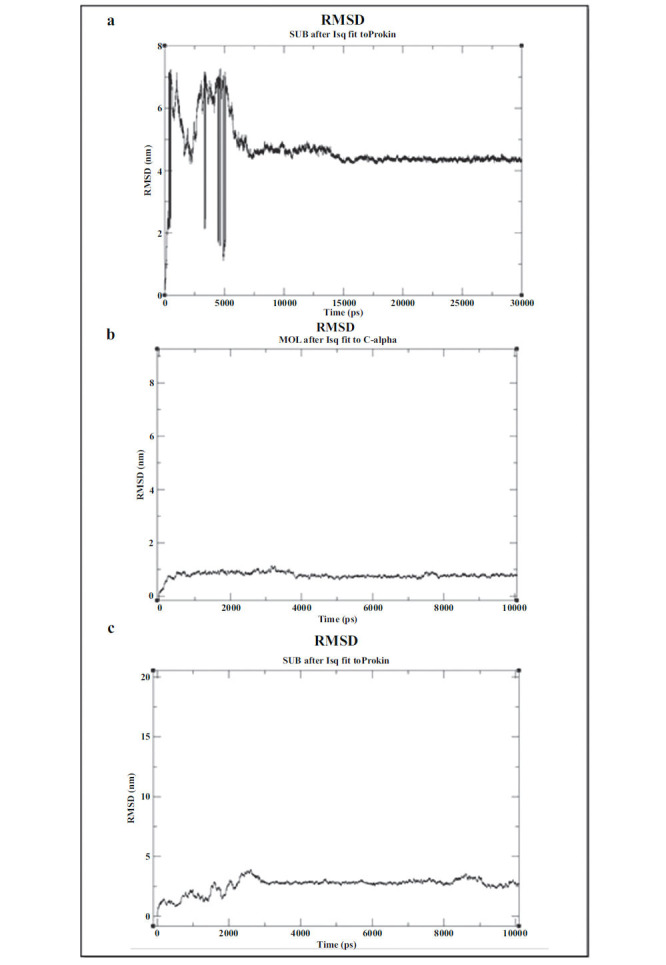
Molecular dynamics simulation of cabozantinib: Stabilized hydrogen bond interactions with toxicity-associated protein. (**a**) AURKB (PDB ID: 4AF3), (**b**) LCK (PDB ID: 3LCK), and (**c**) TEK(PDB ID: 2GY5).

**Fig. (6):**
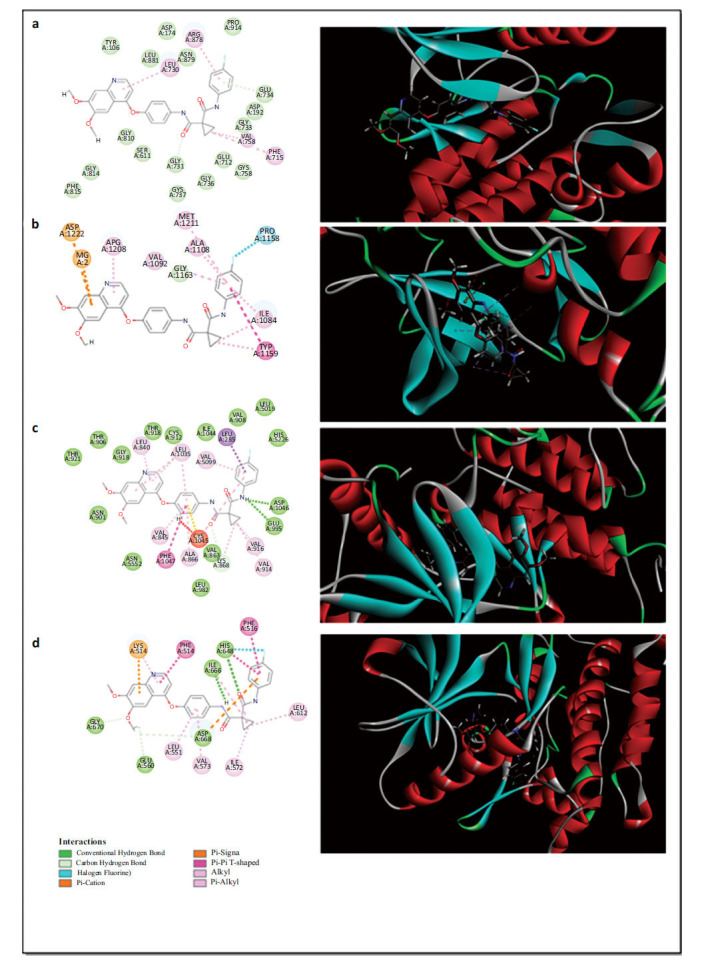
2D (left) and 3D (right) interaction diagrams of cabozantinib with primary renal cancer targets. (**a**) RET (PDB ID: 2IVU), (**b**) MET (PDB ID: 3DKC), (**c**) VEGFR2 (PDB ID: 3VO3), (**d**) Trk-A (PDB ID: 6PL3).

**Table 1:** The screened targets identified through the Venn diagram.

**Public Protein**	**Target**	**PDB ID**
Kinesin-1 heavy chain/ Tyrosine-protein kinase receptor	RET	7DUA, 4CKJ
Serine/threonine-protein kinase Aurora-B	AURKB	5EYK, 4AF3
Vascular endothelial growth factor receptor 2	KDR	3WZD, 5EW3
Tyrosine-protein kinase LCK	LCK	3MPM, 2IIM
Tyrosine-protein kinase TIE-2	TEK	2OSC, 2P4I, 6MWE, 5MYA, 2GY5, 8PLQ
Serine/threonine-protein kinase Aurora-A	AURKA	8JMX, 5DPV
Vascular endothelial growth factor receptor 3	FLT4	6V0X, 4BSK, 4BSJ, 2X1W, 2X1X

**Table 2:** Common toxic side effects associated with key protein targets in renal cell carcinoma treatment.

**Public Protein**	**Common Name**	**Mechanism**	**Toxic Side Effects**
Serine/threonine-protein kinase Aurora-B	AURKB	cell cycle regulation	Myelosuppression
Tyrosine-protein kinase LCK	LCK	co-stimulatory	Immunosuppression
Tyrosine-protein kinase TIE-2	TEK	angiogenesis	Angiopathy

**Table 3:** Results of docking.

**Target**	**Compound**	**Libdockscore**	**Absolute Energy**	**Relative Energy**
4AF3	Axitinib	112.493	129.944	0
	Cabozantinib	126.131	106.287	6.80271
	Lenvatinib	109.084	109.275	1.95811
	Sorafenib	100.969	100.952	5.48012
3LCK	Axitinib	94.5063	134.927	12.2029
	Cabozantinib	87.3385	112.577	14.89
	Lenvatinib	93.4594	113.343	1.05557
	Sorafenib	93.8366	100.604	5.13225
2GY5	Axitinib	104.671	129.944	0
	Cabozantinib	123.702	102.082	3.75174
	Lenvatinib	97.7654	112.934	0.785837
	Sorafenib	86.9655	106.665	11.1934

**Table 4:** Results of Cabozantinib docking.

**Common**	**Target**	**Libdpckscore**	**Absolute Energy**	**Relative Energy**
VEGFR2	3VO3	142.735	104.827	6.49667
MET	3DKC	110.014	102.207	3.87638
RET	2IVU	123.709	98.3305	0
TEK	7LD3	139.86	98.4172	0.0867

## Data Availability

All the data and supporting information is provided within the article.

## LIST OF ABBREVIATIONS

SAR: Structure-Activity Relationship
RCC: Renal Cell Carcinoma
TKIs: Tyrosine Kinase Inhibitors
